# Fractal approaches to scaling transformations to sustainability

**DOI:** 10.1007/s13280-023-01873-w

**Published:** 2023-05-19

**Authors:** Karen O’Brien, Rosario Carmona, Irmelin Gram-Hanssen, Gail Hochachka, Linda Sygna, Milda Rosenberg

**Affiliations:** 1 Department of Sociology and Human Geography, University of Oslo, Blindern, P.O. Box 1096, 0317 Oslo, Norway; 2 Transformative Change Network at University of Oslo, Pedro Torres 460 apt. 405, 7790634 Santiago, Chile; 3 Western Norway Research Institute, Røyrgata 4, 6856 Sogndal, Norway; 4 Forests and Communities in Transition (FACT) Lab, Faculty of Forestry, University of British Columbia, 2424 Main Mall, Vancouver, BC V6T 1Z4 Canada; 5 cCHANGE, Skedsmogata 14, 0655 Oslo, Norway

**Keywords:** Agency, Fractals, Relational paradigms, Scaling, Three Spheres of Transformation, Universal values

## Abstract

Responses to sustainability challenges are not delivering results at the scale and speed called for by science, international agreements, and concerned citizens. Yet there is a tendency to underestimate the large-scale impacts of small-scale, local, and contextualized actions, and particularly the role of individuals in scaling transformations. Here, we explore a fractal approach to scaling sustainability transformations based on “universal values.” Universal values are proposed as intrinsic characteristics that connect humans and nature in a coherent, acausal way. Drawing on the Three Spheres of Transformation framework, we consider how enacting universal values can generate fractal-like patterns of sustainability that repeat recursively across scales. Fractal approaches shift the focus from scaling through “*things*” (e.g., technologies, behaviors, projects) to scaling through a *quality of agency* based on values that apply to all. We discuss practical steps involved in fractal approaches to scaling transformations to sustainability, provide examples, and conclude with questions for future research.

## Introduction

What do we do now? Despite increased attention to the interlinked crises of climate change and biodiversity loss, there is a discouraging lack of results when it comes to meeting internationally agreed-upon goals and targets. Current approaches do not appear to be effective in responding to the scale and urgency of sustainability challenges, and development trajectories are on track to surpass multiple tipping points and planetary boundaries (Steffen et al. 2018; Armstrong McKay et al. 2022). Despite decades of efforts to promote technical, behavioral, and political solutions, humanity’s inability to successfully respond to the scale, magnitude, and scope of sustainability challenges has left many people with a growing sense of despondency and a diminished sense of agency (Bendell 2018; Cunsolo and Ellis 2018). This suggests a need to consider new approaches to conceptualizing, designing, implementing, and scaling sustainability solutions.

The failure of sustainability solutions to deliver results at the scale and speed called for by science, international agreements, and concerned citizens directs attention to the process of scaling. The literature on scaling sustainability solutions points to the need for structural and systemic changes based on new ways of thinking, doing, being, and organizing (Moore et al. 2015; Augenstein et al. 2020; Lam et al. 2020; Schut et al. 2020). In reviewing this literature, Lam et al. (2020) identify various relevant concepts, such as stabilizing, growing, replicating, transferring, spreading, and speeding up. Westley et al. (2014) describe *scaling out* as efforts to replicate and disseminate innovative programs, products, ideas, or approaches to affect a larger group of people or cover a wider geographical area and *scaling up* as efforts seeking to make qualitative policy changes that alter the rules of the game within systems. Moore et al. (2015, p. 74) add the concept of *scaling deep*, referring to the moment when structural changes transform the quality of social relations; this occurs through changes in “people's hearts and minds, their values, and cultural practices.” Others point to the importance of *scaling down*, which recognizes the importance of enhancing local involvement in “non-scalable” projects as an entry point for systemic change (Lampinen et al. 2019).

While there are many theories and frameworks for scaling, sustainability science in general has not reconciled the gap between global-scale problems and local-scale solutions (Shrivastava et al. 2020). Importantly, global *problems* such as climate change have long been recognized to result not only from large-scale systemic changes, but also from the substantive or areal accumulation of many small, local-scale changes (Turner et al. 1990). Yet in striving for global *solutions* to sustainability challenges, there is a tendency to dismiss or downplay the impact of small-scale actions, particularly individual actions. Consequently, the large-scale implications of local, contextualized actions have yet to be understood and activated in current scaling approaches.

One way of addressing this scalar gap is to look more closely at how human agency can transcend scales to connect individual change, collective change, *and* systems change. Human agency can be defined as the capacity to change systems through conscious actions (Westley et al. 2013). Often a distinction is made between individual agency and collective agency, or between everyday agency and strategic or political agency (Otto et al. 2020). While such distinctions are conceptually useful, separating the individual from the collective and the everyday from the political may limit understandings of humanity’s potential to scale sustainability solutions. Also, in focusing on individuals and assumptions about their limited efficacy, there can be a risk of reducing and atomizing change processes, while ignoring their relationship to structural and systemic change.

How can scaling approaches better acknowledge relationships between individual and collective agency, and between local and global solutions? There are several salient points about scaling that are not adequately captured in current frameworks and practices, and they warrant further consideration. For example, the role and influence of human interiority, such as worldviews and values, is recognized by Moore et al. (2015) as important in scaling practices, yet seldom included. Further, the role of values that apply universally to all—such as equity, dignity, fairness, and compassion—has not been considered in scaling sustainability solutions. In short, linear models of scaling have failed to capture the holistic, reciprocal, and entangled ways in which individual actions can generate nonlinear change.

In this perspective, we explore the potential for individual and collective agency to spark, scale, and sustain transformations to sustainability, and we present a fractal approach to scaling transformations based on “universal values.” We start by discussing the challenge of scaling sustainability solutions, describing some of the limitations of current paradigms. Next, we focus on relational paradigms and consider what they offer to understandings of scaling. Drawing on the Three Spheres of Transformation framework, we then describe how a fractal approach to scaling that is grounded in universal values can simultaneously work across the practical, political, and personal spheres (O’Brien and Sygna 2013). We discuss *fractal agency* as a pivotal piece for scaling sustainability solutions, and outline some steps for putting this relational approach into practice. We conclude by suggesting some future areas for research on fractal approaches to scaling sustainability.

The fractal metaphor presents a different and potentially more useful way to conceptualize scaling sustainability transformations and to reconcile local- and global-scale solutions. Fractal approaches shift the focus from scaling through “*things*” (e.g., technologies, behaviors, projects) to scaling through a *quality of agency* based on values that apply to all, such as oneness and integrity. In practice, scaling through fractal agency involves building capacities of individuals and groups to generate context-specific patterns based on universal values that recursively repeat at all scales to create a world where people and planet can thrive.

## Scaling sustainability

Scaling has been described by Schut et al. (2020, p. 1) as “the adaptation, uptake and use of innovations such as practices, technologies, and market or policy arrangements across broader communities of actors and/or geographies.” It is related to the geographical concept of scale, which can mean level, size, or relation. However, in practice, scales are never separate or discrete (Howitt 1998; Jones 1998). There have been extensive academic discussions over the past decades about the concept and significance of scale, with researchers arguing that scale is a construction, an abstraction, a relation, a process, and a shaper of social power relations, rather than a “thing” in itself (Howitt 1998; Paasi 2004; Swyngedouw 2004). Acknowledging the limitations of “[t]he tired dualisms and rigidities of conventional thinking,” Howitt (1993, p. 40) introduces a relational conception of scale that recognizes its embeddedness in the dynamics of social life. Marston et al. (2005) have argued for eliminating the concept of scale from human geography, opening up for alternative scalar imaginaries, including flat ontologies that represent self-organizing, emerging systems that actualize as temporary sites where the social unfolds.

### Solutions that scale

Despite academic debates challenging the concept of scale, sustainability science has emphasized the need to scale technical and managerial solutions to global challenges (Cuéllar-Gálvez et al. 2018; Ivory and MacKay 2020). Additionally, a growing number of sustainability scholars argue that it is also necessary to scale contextualized behavioral changes (Carmi et al. 2015; Newell et al. 2021). The scaling of technical and managerial solutions and behavioral changes is often based on the idea that once a large number, critical mass, or social tipping point is reached, broader systems will respond (Bentley et al. 2014; Milkoreit et al. 2018). However, reducing systemic problems such as climate change to scaling technical, managerial, and behavioral changes often marginalizes or excludes serious engagement with structural analyses, which in turn risks perpetuating exploitative and oppressive relations (Blythe et al. 2018; Gram-Hanssen et al. 2022). For everyday actions to generate and support an equitable and thriving world, structural causes of the problem must also be addressed, thus attention must also be placed on social and cultural norms, rules, regulations, institutions and paradigms.

When the structural causes of problems are not addressed and people are nudged or simply told to abide by certain solutions, responses often produce rebound effects, negative spillovers, and moral licensing (Schubert 2017; Newell et al. 2021). The imposition of change on others tends to be ineffective in the long term and contentious in the short term, creating resistance and polarization and raising critical questions about justice and power dynamics (Blythe et al. 2018; Bennett et al. 2019). Furthermore, solutions that are successful in one context may not always be relevant in others. In contrast, changes that are chosen or embraced by people based on what they deeply care about for themselves and others, and expressed in a context-sensitive and relevant manner, are likely to be more effective and enduring (Sharma 2017; Hochachka 2019). Finally, the scaling of transformations cannot be reduced to cause–effect analyses or linear assumptions about the dynamics of individual–collective interactions (Moore and Milkoreit 2020). Today’s urgent global environmental challenges call for scaling sustainability solutions in an equitable, inclusive, and nonlinear manner (Powell 2019).

### Structures and (qualities of) agency

Social theorists have long debated the relationship between structure and agency. Most of these debates recognize structure as recurrent patterns that influence or limit the choices and opportunities available to agents, and agency as people’s capacities and power to act deliberately. Many theorists emphasize the interdependence between structure and agency (Wendt 2015). However, insufficient attention has been given to the *quality* of agency that is needed for shifting structures *and* scaling equitable and enduring solutions to global challenges. An exception is sociologist Archer (2000), whose morphogenetic approach emphasizes that the capacity of agency to modify the structure is anchored in the notion of reflexivity. Archer (2000, p. 152) explains that individuals become reflexive through the emergence of personal identity, consisting of “the self, formed through our embodied relations with the natural world.” The development of self-consciousness makes it possible for individuals to consider the society around them and their actions within it. Emphasizing this qualitative dimension to agency recognizes that not all expressions of agency benefit the whole, and that partial responses are likely to contribute to siloed or misaligned policies and practices. As Zanotti (2019) writes, agency without an ethos of responsibility is more likely to create unintended consequences.

Emphasizing that physics has informed understandings of the modern social world, international relations theorist Wendt (2015) argues that most theories of structure and agency are inherently classical. By classical, he means that they are based on subject–object, mind–body, and human–nature dualisms. Taking instead a relational, quantum perspective, Wendt (2015) views structures as the continuous collapse of a wave of potential into a single, classical outcome. Often this collapse reinforces the well-worn grooves of current action-logics and the systems they perpetuate. The potential to consciously generate new patterns is associated with individuals and their entangled and reflective agency. To realize an equitable and sustainable world that benefits all requires *qualities of agency* that are grounded in oneness and a sense of responsibility for the whole (O’Brien 2021).

Recently, there has been growing attention to the interior and subjective dimensions of transformative change, including the importance of values and personal transformations (Horcea-Milcu et al. 2019; Wamsler et al. 2021). However, personal transformations alone are seldom sufficient to transform inequitable and unsustainable systems and structures that are maintained by power, politics, privilege, and vested interests (Blythe et al. 2018). Recognizing that integrative approaches can contribute new insights to existing sociological ideas about structure and agency, an important question for research and practice is how conscious, value-based changes made in diverse contexts by individuals or collectives can transform systems in ways that are measurable and significant to global sustainability.

## New paradigms for scaling transformations

To link individual change, collective change, and systems change calls for a shift in the ways that we think about scaling change, i.e., a paradigm shift. Paradigms refer to the concepts, metaphors, and thought patterns that form the basis of scientific theories and methods. Paradigms inform how different groups understand the world and how they organize society. The paradigms that are deemed legitimate or valid in any particular context are established and maintained by actors situated within hegemonic cultural groups, and their discourses tend to influence both how they perceive problems and prioritize solutions (Leichenko and O’Brien 2019). Consequently, paradigms are considered a powerful leverage point for systems change (Meadows 1999).

The very paradigms that enabled the human species to become a global force through industrialization and intensive resource extraction are the same ones underpinning currently unsustainable systems, structures, relations, and responses (Lövbrand et al. 2015; Whyte 2020). In the context of global science and policy, a Western positivist paradigm has long been prioritized, whereas others have been considered “alternative” or irrelevant (Sundberg 2014; Wendt 2015; Todd 2016). While eventually an integration of paradigms may be needed, sustainability science research has been placing increasing attention on relational paradigms that diverge from positivism and take into consideration the entangled state of humans and nature and the nonlinear dynamics of change (West et al. 2020; Walsh et al. 2021).

### Relational paradigms

Relational paradigms refer to ontologies, epistemologies, and ethics that do not presuppose subject–object and nature–culture binaries (Walsh et al. 2021). In contrast to classical approaches to social change that conceive of individuals as entities separate from each other and their environment, relational paradigms are based on principles of interconnectedness, oneness, and entanglement. Relational paradigms broaden conceptualizations of being in the world and expand the role of agency, recognizing that it can be distributed across networks, configurations, and assemblages (West et al. 2020). Such paradigms have a long history within Indigenous thinking and academic scholarship. As Wildcat (2005, p. 433) explains, “the indigenous cultures emergent from many places on the planet operate on assumptions, paradigms, and a unique sense of history and time that contradict Western notions.”^1^ For instance, Indigenous cultures such as the Yup'ik of southwestern Alaska and the Guna of Panama do not consider individuals as isolated and separate. Instead, both humans and non-humans are understood to be inherently connected with each other and with nature, which in turn supports an understanding of individual and collective agency as co-arising (Apgar et al. 2015; Gram-Hanssen 2021). Relational paradigms have received considerable attention in recent years through assemblage theory, actor network theory, new materialism, quantum social theory, agential realism, and other approaches within the social sciences and humanities (Latour 1992; Barad 2007; Alaimo 2010; Coole and Frost 2010; Wendt 2015; Cadena and Blaser 2018; Escobar 2020). Yet there has only been limited attention to what relational paradigms mean for understandings of how to scale transformations (Grandin and Haarstad 2021).

### Relational scaling

Within a relational paradigm, successful scaling calls for a shift in how individuals relate to themselves, to each other, to nature, to systems, and to change itself (O’Brien 2021). Grandin and Haarstad (2021) draw attention to the cross-scalar and inter-connected character of processes and agencies and the need to disrupt linear and hierarchical understandings of scale and scaling. Relational paradigms highlight the importance of “small” initiatives, while at the same time calling for contextualized structural evaluations (Gibson-Graham 2008). Transforming inequitable and unsustainable relationships in society requires disrupting old patterns and generating new, qualitatively different ones (Sharma 2017).

Relational approaches may inform new, empowering, and actionable practices for scaling sustainability, particularly when they are based on values that apply inclusively and recursively to the whole. Values are increasingly recognized as essential to sustainability, including both relational and transcendental values, i.e., those that “transcend specific situations and guide selection or evaluation of behavior and events” (Horcea-Milcu et al. 2019, p. 1426). There is considerable research on the role of values in environmental behavior, as well as agreement that values predict ecological behavior (Karp 1996; Steg 2016; Thierman and Sheate 2020). As the IPBES Values Assessment (2022, p. 31) notes, “Transformative change toward sustainability can be facilitated through policies designed to incorporate sustainability-aligned values into established social conventions, norms, and legal rules that shape human–nature relations.” Recognizing the importance of values, we next present a fractal approach to scaling transformations based on universal values.

## Fractal approaches to scaling

Fractals are self-similar patterns that repeat across many scales, such that small parts of an object look similar to the whole (Mandelbrot 1977). Fractal patterns are simple to generate, yet dynamic and infinitely complex. By repeatedly applying a rule, definition, procedure, or principle to successive results, resulting patterns add a fractal dimension that does not adhere to traditional scaling taxonomies. Fractals that are infinitely self-similar can be produced mathematically, whereas fractal-like patterns that are finite are common in nature and can be seen in spirals, branches, or patterns such as in beehives, leaves, river systems, and coastlines (Mandelbrot 1982). The idea of patterns that recursively repeat is not limited to mathematics and nature; fractals have been applied to society as well.

### Social fractals

Social fractals can be described as self-similar patterns that repeat themselves across a range of structures at different scales, extending from small social interactions to large national and international institutions. They can be generated by principles, values, ideas, initiatives, or endeavors that are designed with the same characteristics desired for the whole (Sharma 2017). Perey (2014) presents fractals as a scale-independent categorization that describes rules and principles that organize a system, and he points out that it is a useful metaphor for sustainability solutions. Metaphorically, the concept of fractals has been applied to social processes to describe unique, context-specific patterns that represent multiple versions of the whole, or “a whole in microcosm” (Downton 2008, p. 27). In describing a fractal sociology, Jensen (2007, p. 838) considers that “all social events are on the same level, in the sense that each set of events can be described as equally complex regardless of their putative fit into a micropicture or macropicture.”

Bernstein and Hoffman (2019) use a fractal lens to analyze decarbonization, suggesting that a global fractal metaphor can help to disrupt carbon lock-in at multiple levels and scales. They argue that actions in one sector, jurisdiction, or society interact with similar patterns elsewhere (Bernstein and Hoffmann 2019, p. 920). Fractal politics has been described by Adnan (2021, p. 119) as “the sense that similar developments are occurring in similar patterns all over the world without any centralized agency.” She recognizes the revolutionary potential of breaking the “trances of disconnection,” and calls for working with a fractal sensibility, rather than a linear one (Adnan 2021). A fractal sensibility contrasts with the current discourse on sustainability solutions, which struggles to reconcile the distinction between top-down approaches (e.g., international goals, targets, strategies, and initiatives) and bottom-up approaches (e.g., the many local, national, and regional efforts that are place-based, contextual, and responding to multiple dynamics) (Wilbanks 2007; Aguiar et al. 2020).

Perey (2014, p. 216) emphasizes that fractals are more than just metaphors or tools of observation and measurement: “they are also tools of intervention into the dynamics of social systems.” For instance, in looking at the intrinsic properties that produce scaling behavior, Frankhauser (2021) explores how the scaling properties of fractal geometry can help to design development scenarios that optimize the spatial organization of metropolitan areas, thus contributing to sustainability. Fractal principles are used to understand and measure organizational attitudes and their contributions to “a global concerted move toward sustainability” (Canto de Loura and Dickinson 2018, p. 32). A fractal approach, as proposed here, is designed to “move the whole” by generating patterns of change that scale.

### Fractal agency and universal values

The ability to generate coherent patterns based on values that apply to all can be understood as “fractal agency.” Fractal agency recognizes that humans are connected from the micro to the macroscale through entangled patterns and relationships (McCaffrey and Boucher 2022). This connection depends on both the moral integrity of individuals and their capacity to connect values with action (van der Werff et al. 2013), as well as on the unique conditions provided by the context (Steg 2016). Both agency and an ethos of responsibility for people and the planet are linked to values.

The IPBES Values Assessment points out that “Values-centered concerted actions by social actors are needed to achieve shared visions to revert the biodiversity crisis and navigate toward more sustainable and just future” (IPBES 2022, p. 44). Extensive research suggests that values that are considered universal promote a more egalitarian worldview that encourages more ethical decision-making, allows overcoming conformity to the unsustainable status quo, and is associated with a greater sense of wellbeing, which, in turn, stimulates more sustainable behaviors (Hedlund-de Witt et al. 2014; Pfattheicher et al. 2016; Kasser 2017). Universal values are considered critical to the success of the U.N. Sustainable Development Goals (SDGs); they “enable the SDGs to be truly transformative, by placing the person and their inherent dignity at the heart of development efforts, empowering all people to become active partners in this endeavor” (UN Sustainable Development Group 2023). Greater understanding of what conditions best support such values to develop and become enacted is an important area for further research.

We interpret the proposition of universal values as *intrinsic and shared qualities and characteristics that connect humans and nature in an acausal, coherent manner.* Here, acausal describes a connection that is innate and entangled, and coherent refers to forming a whole. As opposed to culturally determined values, universal values “transcend religious tenets, norms, and other social diktats” (Sharma 2017, p. 3). However, many world philosophies and religions do include similar deep, basic (meaning primordial and self-inherent) values for humans and other species, for example Trungpa’s (2007, p. 31) “basic human wisdom.” Universal values are partially aligned with relational values, which aim to overcome dichotomies between intrinsic values (for nature’s sake) and instrumental values (for human’s sake) by focusing on the wellbeing of human–ecosystem relationships (Chan et al. 2016, 2018). While relational values emphasize the relationship itself, which often varies across contexts, universal values are those that apply to all human and non-human life, across all contexts, with no-one excluded (Sharma 2017). The interpretation of universal values can also be distinguished from Schwartz’s (1994) work on universal values. According to Schwartz (1994), certain values are considered universal because they are *held* by individuals across many countries and cultures. Such basic or universal values include security, achievement, and benevolence. In contrast, universal values as used here are those values that *inherently apply* to all humans and non-humans, regardless of whether they are consciously held or recognized.

The proposition of universal values suggests that as people recognize and act from intrinsic qualities and characteristics that connect humans with each other and with nature, their actions may more impactfully align with sustainability. This deep sense of interconnectedness among and between humans and nature aligns with relational paradigms that transcend subject–object and mind–body dualisms (Hertz et al. 2020). Interpreted in this way, universal values emphasize that individuals, collectives, and systems are not separate and discrete, but connected, entangled, and intra-acting, and that all actions inevitably have non-local repercussions, even if these are subtle and, in many cases, beyond conscious awareness (O’Brien 2021). Such values that recognize the interdependence of the parts (i.e., ourselves, all people, all beings, forms, processes) with the whole can be considered fundamental to an equitable and sustainable world.

## Fractal scaling in practice

Fractals represent patterns with integrity, with each unique fractal contributing to a larger field of change. Achieving an equitable and sustainable future for all in practice demands a reflexive, values-centered approach to agency, a *qualit*y *of agency* that can strategically transform systems and relations in an integrative manner (O’Brien 2021). Fractal agency can strategically scale and generate ripples and cascades that amplify, generating a “spiral” of sustainability (Newell et al. 2021). To illustrate what fractal approaches to scaling might look like in practice, we draw on the collective experience of the author-team from both empirical and theoretical research on the topic of scaling sustainability, including quantum social change (O’Brien 2016, 2021), integral and integrative models (Hochachka 2009, 2021, 2022; O’Brien and Hochachka 2010), Indigenous Peoples’ perspectives (Carmona 2023), and relational values and paradigms (Gram-Hanssen 2021; Rosenberg 2022). We also draw on the strategic and practical “conscious full spectrum response” model for transformations, developed by Sharma (2007, 2017) as part of her work within the United Nations to deliver results at scale (McElhenie 2005; Sharma et al. 2005).

To bring these insights together, we draw on the Three Spheres of Transformation framework, an actionable framework for designing and implementing transformations to sustainability (O’Brien and Sygna 2013). The framework emphasizes relationships among the practical, political, and personal spheres of transformations and has been used in research on a number of thematic topics relevant to sustainability, including climate change (O’Brien 2018; Jacobson et al. 2020; Thiermann and Sheate 2020; Hochachka et al. 2022). Three Spheres of Transformation describes a way to engage with fractal approaches to scaling sustainability, as explained below.

### The Three Spheres of Transformation

**Fig. 1:**
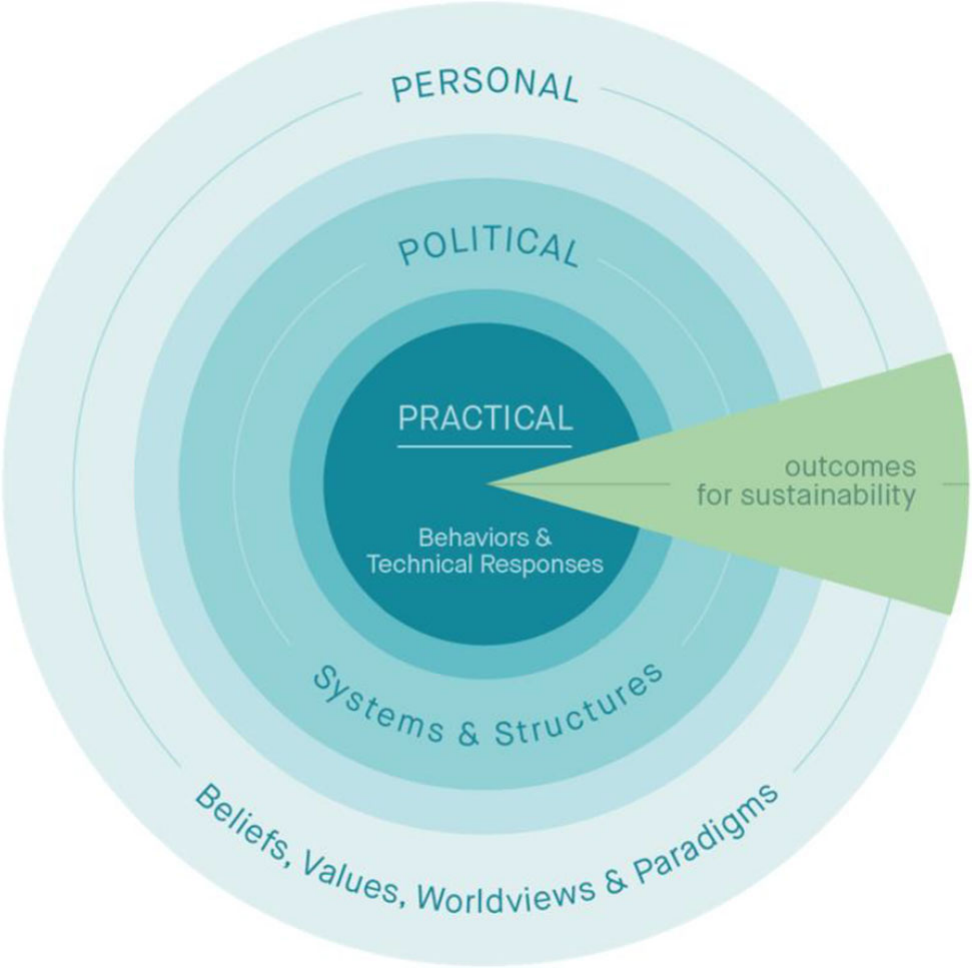
Three Spheres of Transformation. *Source* O’Brien and Sygna (2013), based on Sharma (2007)

The Three Spheres of Transformation is a heuristic that describes the process of transformation in a simple yet comprehensive manner (Fig. 1). Although the three spheres are connected and interrelated, actions to scale sustainability seldom consider the practical, political, and personal spheres together, i.e., as unified and co-arising whole. As we explain below, scaling sustainability transformations depends on engaging with all three spheres in a holistic manner.

#### Practical sphere

The practical sphere of transformation focuses on actions and interventions that directly contribute to measurable outcomes. Transformations in the practical sphere tend to emphasize technical approaches to change, e.g., new technologies and behaviors. These measures demand investment, enhanced knowledge, and sometimes nudging. Many hope that a proliferation of such innovations and programs will lead to “win–win–win” situations that scale to simultaneously address climate change, biodiversity loss, and the SDGs (van der Waal et al. 2021). Nevertheless, these measures often overlook the power dynamics within economic, political, and socio-ecological systems and the diversity of beliefs, values, and worldviews that influence these dynamics. As a result, when it comes to scaling, interventions that only engage the practical sphere tend to deliver disappointing results (Thumm and Perl 2020; Bain 2021; Hochachka et al. 2022).

#### Political sphere

The political sphere refers to the structures and systems that influence or govern how society is organized, including which values are prioritized, whose goals are pursued, how solutions are negotiated, decided upon, and implemented, and by whom. This recognizes that social and cultural norms, institutions, regulations, and incentives can facilitate or hinder the scaling of practical changes, and acknowledges that power, vested interests, and competing commitments influence decision-making processes. Those in positions of power and privilege often uphold and protect existing systems, resulting in inertia, lock-in, polarization, antagonisms, or prolonged conflicts. Scaling through the political sphere requires recognizing that transformations create resistance that is often linked to diverse views of what is important, why, and for whom.

#### Personal sphere

The personal sphere refers to the individual and shared beliefs, values, worldviews, and paradigms that influence how people perceive, define, or constitute systems and structures, and how they relate to each other and nature, including non-humans. These subjective dimensions also define how issues of agency and causality are perceived and addressed. This influences whether structures and systems are seen as fixed and “given,” or as capable of changing. It also includes what matters to people, both individually and collectively. The personal sphere informs how the scaling of transformations to sustainability is conceived and ultimately realized. In the following section, we examine how fractal agency can play a pivotal role in scaling change and provide examples.

### Fractal agency as a pivotal piece

Fractal agency can be thought of as both a quality and a capacity to generate patterns that are context-specific yet aligned to strategically transform inequitable and unsustainable relationships. This recognizes that thoughts, ideas, words, metaphors, decisions, conversations, actions, and agency generate entangled patterns that scale (O’Brien 2021). Rather than reserving strategic action for those at the top of political, business, or organizational hierarchies, fractal agency describes a capacity that all people can access and implement, independent of position, degree, role, experience, or authority (Sharma 2017). This is an empowering approach to scaling that transforms disempowering relationships. The past is full of examples of seemingly ordinary human beings transforming the course of history (Solnit 2004), and during the last few centuries, it has become clear how the scope of human actions has geological repercussions with profound implications for social transformations (Olsson et al. 2017). In a similar vein, fractal agency recognizes that *everyday actions matter across scales*.

**Fig. 2:**
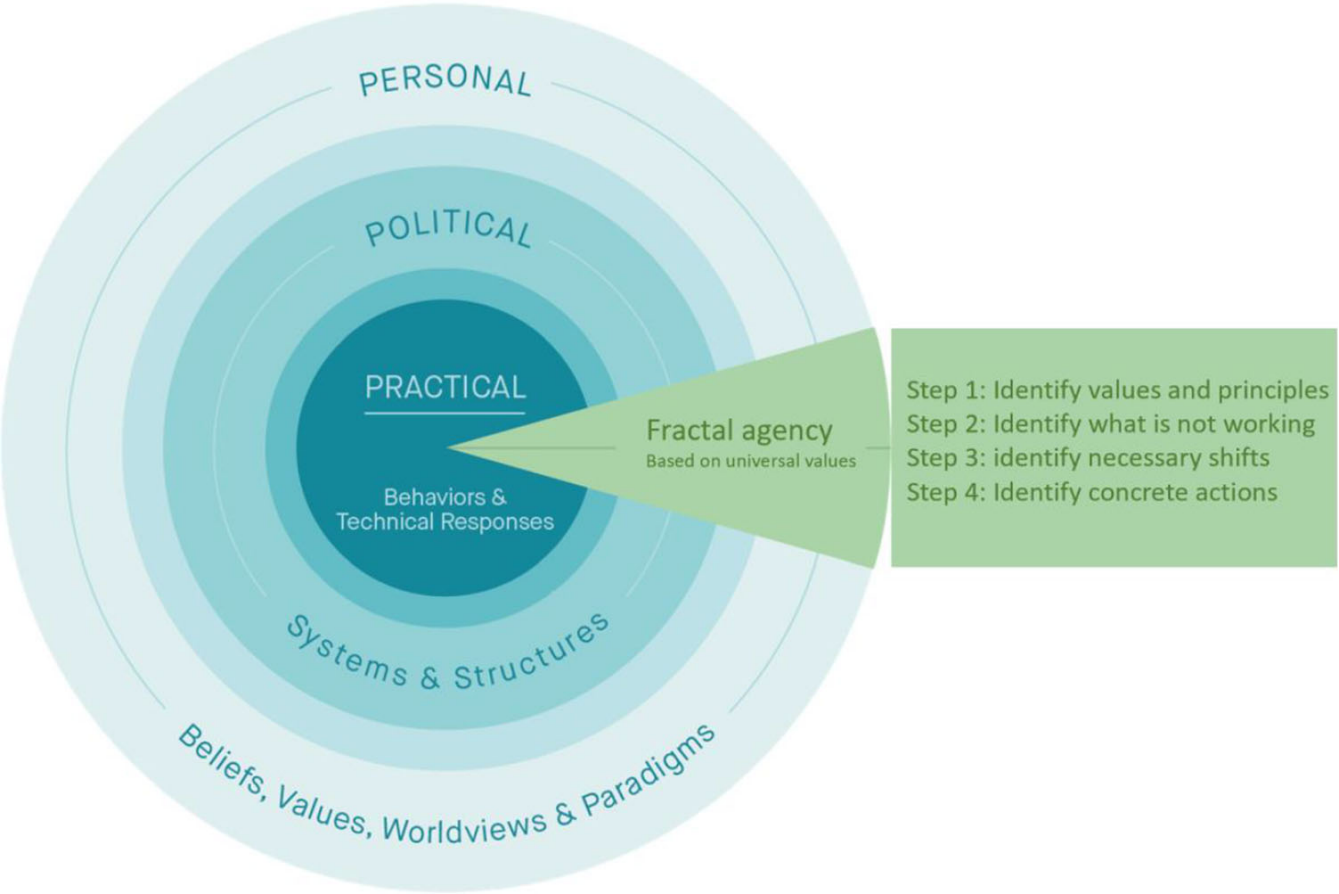
Modification of the Three Spheres of Transformation, emphasizing the role of fractal agency in scaling sustainability (based on Sharma 2007, 2017; O’Brien and Sygna 2013)

Below, we describe four steps central to fractal agency (see Fig. 2). The four steps are not sequential, and more like a dance that takes us back and forth across the three spheres, touching simultaneously on practical, political, and personal dimensions of transformative change and recognizing them as an integrated whole.

**Step 1 - Personal Sphere:** A starting point is for each of us individually to recognize the universal values that are important to us (and relationally, to all), i.e., *what we deeply care about for all*. It also involves identifying what *principles* we collectively agree to follow in our work, projects, or initiatives.

**Step 2 - Practical Sphere:** Next, we express in words what problem we want to contribute to solving, and *how the problem shows up* in practice, i.e., what’s not working and which current practices are hampering our ability to create a future that works for everyone. Based on this, we can decide *what visible changes and measurable results* we would like our actions to generate.

**Step 3 - Political Sphere:** We then identify which current systems maintain the status quo, and the *cultural and systemic shifts* that need to happen for measurable results to materialize in an equitable and sustainable manner, i.e., which norms, rules, regulations, institutions, and narratives need to shift, with an emphasis on a directional shift *from* the status quo *to* something new.

**Step 4: - Being in Action:** We then identify and take *specific actions* based on universal values, both in the short term and long term. This includes everything from organizing meetings to launching new initiatives and establishing new protocols based on universal values that shift systems in the political sphere, and produce results we wish to see in the practical sphere.

Fractal Agency is a pivotal piece in all four of the above steps that aligns the personal, political, and practical spheres of transformation. Integrating these steps connects *who we are being* and *what we are doing*, such that the root factors of problems are addressed in a manner that creates equitable and sustainable social change across scales (Sharma 2017). While systems and cultural shifts are thought to take decades to be accomplished, a fractal approach allows for deliberately and strategically creating and embodying the future right now, in the present (Trott 2016; Sharma 2017; O’Brien 2021). In a strategic and enactive manner, fractal agency can shift systems and cultures that keep societies locked into inequitable and unsustainable patterns and relationships.

**Fig. 3:**
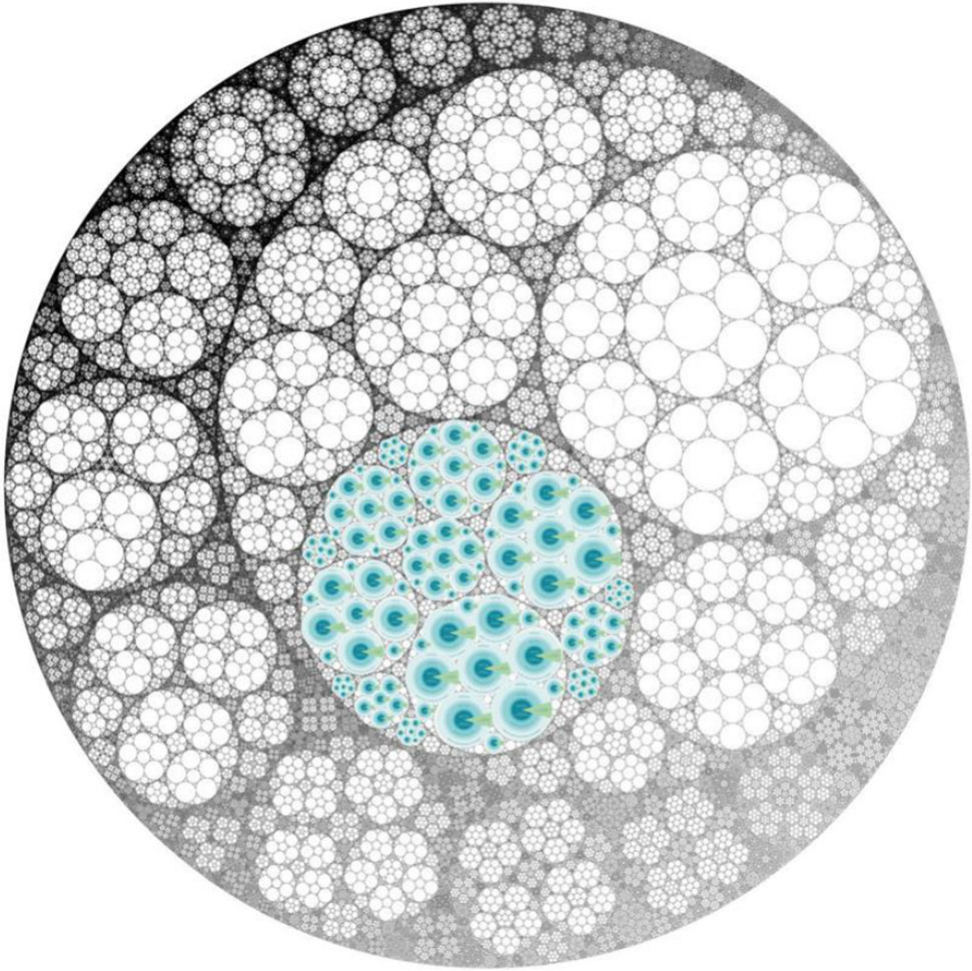
The Three Spheres of Transformation as a fractal approach to sustainability (depicted here within an Apollonian Gasket fractal)

A fractal approach to scaling transformations is a process that creates a field of change based on integrity or wholeness, where each unique action or initiative nurtures values of relationality, responsibility, reciprocity, and redistribution (Harris and Wasilewski 2004; Gram-Hanssen et al. 2022). Projects and initiatives that address sustainability are likely to be diverse and context-specific, but when agency is grounded in universal values, self-similar patterns transform the larger field (see Fig. 3). Though innate to all humans, fractal agency is a practice that takes awareness, reflexivity, humility, and courage. By transcending dualisms, fractal agency disrupts fragmenting patterns that structure, systematize, and perpetuate inequities and unsustainability. This is not to naively believe that exclusionary, oppressive, and hierarchical patterns and relationships will simply disappear. Instead, it offers a strategic approach to overcoming polarized and fragmentary views that reinforce such relationships, by recognizing that universal values apply to all, and that all actors and agents have a capacity to generate fractal patterns that shift relationships, cultures, and systems.

### Examples of fractal approaches to scaling

**Fig. 4:**
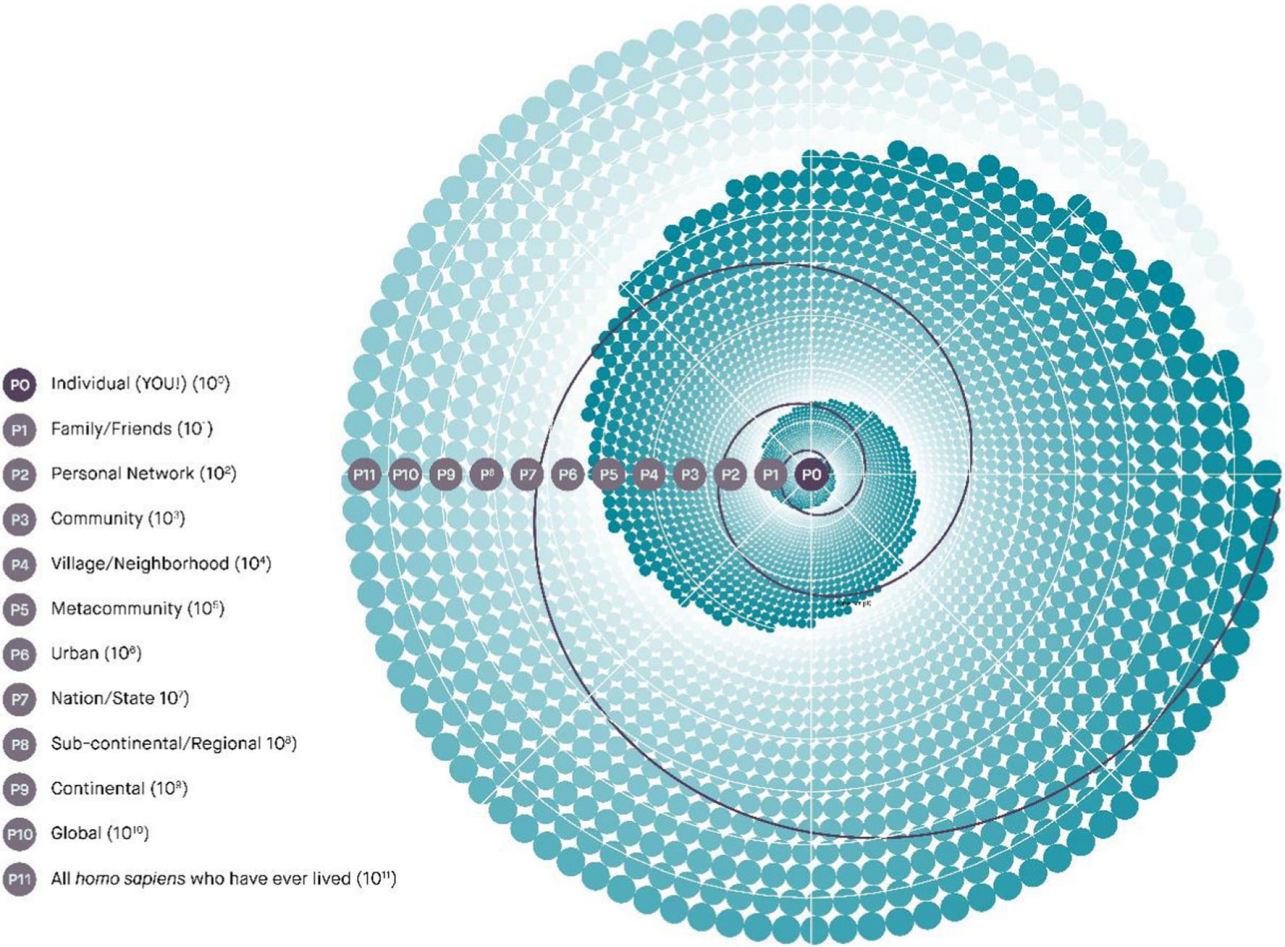
Powers of 10 Fractal Framework, inspired by Bhowmik et al. (2020). *Source* O’Brien (2021)

A fractal approach to scaling has the potential to promote “virtuous cycles” of entangled change at multiple scales (Power 2016). Drawing on Bhowmik et al.’s (2020) Powers of 10 framework, fractal agency recognizes that individuals can influence family, friends, communities, villages, neighborhoods, metacommunities, and so on, up to continental and global scales. These relations are represented in the “Powers of 10 Fractal Framework” depicted in Fig. 4 (O’Brien 2021). Here, the self-similar property that extends over many scales is not a material property, but a quality of agency. Fractal agency itself can be considered scale free, but it generates impacts across scales.

What do fractal approaches to scaling look like in practice? Illustrative examples of fractal approaches to scaling transformations to sustainability can be challenging to identify because, in practice, most actions and initiatives fragment when universal values are replaced by disempowering ideologies or “isms” (e.g., sexism, classism, racism, anthropocentrism). However, Sharma (2017) provides some examples of this approach based on decades of work across diverse sectors, including with the U.S. National Park Service. As described by Gallo (2014), a three-spheres-like approach has been used in various programs, all of which focus on creating and maintaining thriving communities and a healthy environment while demonstrating the benefits of national parks and highlighting actions that make a difference.

The importance of generating fractal patterns based on universal values can also be viewed through research of land use change. A case study of quality coffee production in Burundi shows how a group of actors consciously working from universal values supported the wellbeing of a local coffee growing community, while also influencing the global value chain for coffee (Rosenberg 2022). The coffee company’s intentions to make an impact on sustainability were grounded in the universal values of care and dignity. Held in a relational manner, these values were present in everyday interactions, policy design and implementation, and funding and outreach programming. With values as a foundation, this group of actors simultaneously engaged with the personal, political, and practical spheres of transformation. Agency based on universal values helped configure material outcomes, such as increased coffee yields and quality, and shift land use toward more effective and sustainable practices, which directly contributed to more just climate change adaptation. The study also found that these new patterns were challenged by existing power dynamics, political structures, and institutional contexts upheld through values of authority and wealth, which constantly worked to undermine ongoing transformations. Rosenberg (2022, p. 531) found that “values are not only determined by the context within which they arise but also configure the unfolding materiality moment by moment.”

A fractal approach to land use change is not unique to Burundi. For example, in their study of the role of deep values in curbing tropical deforestation in the Amazon, Russo Lopes and Bastos Lima (2023, p. 216) emphasize their potential to transcend “us” and “them” dichotomies and conclude that “espousing and acting on certain values can thus be a form of contesting dominant agendas and paving the way for sustainability transformations.” When changes are enacted based on values that apply to all, fractal patterns may create ripples that transform land use patterns and contribute to global sustainability. Applying a fractal lens to scaling transformations can also complement a resilience lens, which recognizes the limitations of “global versus local” framings and embeds principles of resilience to the management and governance of food systems at all scales (Wood et al. 2023). Fractal agency, which involves reflexivity and learning, can contribute to resilience by building capacities to self-organize, nurture diversity, and both unravel undesirable systems and generate new ones at all levels to promote sustainability transformations (Folke et al. 2021).

## Concluding reflections

Fractals are unique, yet when aligned with values such as equity, dignity, and compassion they generate patterns that support the emergence of a “field” of sustainability transformations. A fractal approach to scaling transformations highlights the potential for individuals and groups to work across the practical, political, and personal spheres of transformation. Individuals and groups can source and enact contextual solutions to sustainability that have the potential to scale. To do this, the gap between local- and global-scale solutions requires shifting from scaling technologies, behaviors, and projects to building and activating the agency and capacities of individuals and collectives to transform systems and cultures at scale.

The global sustainability crisis has been produced by a combination of mindsets, systems, and actions that have regarded humans and nature as separate. Throughout history, new systems have been established by individuals and groups that recognize and consciously engage with politics based on values that recognize humans as being part of a larger whole. Fractal agency is based on a recognition that every activity and intervention can contribute to transforming the whole. Fractal approaches to scaling are enactive and recognize that the future is generated day by day, word by word, conversation by conversation, and action by action, rather than through partial and exclusive solutions applied at one scale or another. In contrast to an abstract thought-experiment, fractal agency works through reflexivity and practice. Here we have identified the central steps involved in embodying fractal agency, using the Three Spheres of Transformation as a framework to structure strategic and deliberate engagement with transformative change. How these steps are identified and activated will depend on each context and circumstance.

A fractal approach to scaling introduces new research questions and opportunities for field-based empirical studies. For example, research on scaling transformations to sustainability could collect context-specific examples of how fractal agency contributes to scaling. Empirical studies can be designed to monitor and measure the experiential aspects of fractal agency to gain a better understanding of its scaling effects. To help to test and strengthen the theory *and* practice of transformation, research can explore how people develop and embody universal values, and the role that cultural interpretation plays in the enactment of such values. Finally, new metrics can be developed and evaluated to document the potential for fractal agency to scale.

This approach offers a compelling alternative for addressing scaling dilemmas in sustainability transformations. In contrast to reductionistic approaches, it offers a holistic conceptualization of the relationship between us and others, mind and matter, humans and nature, and subjective and objective perspectives. Fractal approaches reimagine the links between individual and collective agency and “big” or “small” solutions. Instead, they recognize the relationships between individuals, collectives, and systems as co-arising through entangled patterns that replicate and interact across scales (O’Brien 2021). This is an emancipatory approach that can transform institutions and social structures in a manner that substantially reduces human suffering and expands the possibilities for human and planetary flourishing. This paradigm shift, if circulated, diffused, and reiterated through education, capacity development, policy initiatives, and media may generate transformative change that adequately responds to the breadth, depth, and scale of current challenges.

## Footnotes

1 While Indigenous knowledge systems have been increasingly recognized within both science and policy platforms, they tend to be compartmentalized and recognized only regarding some specific practices that align with hegemonic environmental agendas, dismissing the values and worldviews that underpin them which can potentially contribute to rethinking the social and cultural causes of climate change, biodiversity loss, and other sustainability challenges (Carmona et al. 2022).
